# Descriptions of six new species of *Phaonia* Robineau-Desvoidy (Diptera: Muscidae) from China

**DOI:** 10.1093/jis/14.1.132

**Published:** 2014-10-01

**Authors:** Wan-Qi Xue, Hua Rong, Jing Du

**Affiliations:** Institute of Entomology, Shenyang Normal University, Shenyang 110034, P. R. China

**Keywords:** *fuscicoxa-group*, *barkama-group*, key

## Abstract

This paper provides diagnoses and keys to species in the *Phaonia fuscicoxa* -group and the *Phaonia barkama* -group from China; describes six new species, namely *Phaonia subfuscicoxa* Xue and Rong, **sp. nov.** , *Phaonia hypotuberosurstyla* Xue and Rong, **sp. nov.** , *Phaonia caesiipollinosa* Xue and Rong, **sp. nov.** , *Phaonia daliensis* Xue and Du, **sp. nov.** , *Phaonia quadratilamella* Xue, **sp. nov.** , and *Phaonia maoershanensis* Xue, **sp. nov.** We report the distributions and provide notes on the affinities of known species. 
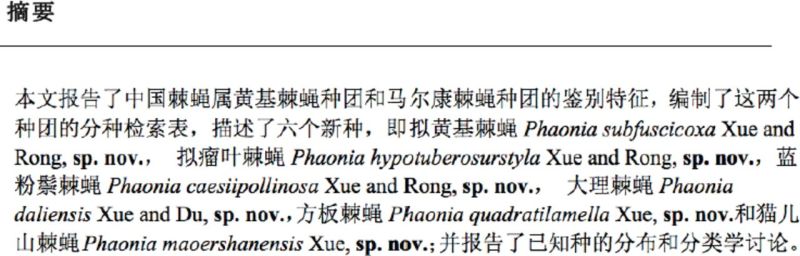

## Introduction

*Phaonia* Robineau-Desvoidy is the most species-rich genus in the Muscidae (Diptera), comprising 806 species in the world, of which 377 species have been described from China. During field collection, some new species of the *fuscicoxa* and *barkama* groups were discovered. The *Phaonia fuscicoxa* species group and the *Phaonia barkama* species group belong to the genus *Phaonia* Robineau-Desvoidy. The *Phaonia fuscicoxa* species group has 23 known species in China, including two new species described in this paper, and the *Phaonia barkama* species group has 12 known species in China, including four new species described in this paper.

## Materials and Methods

Descriptions were based on adult flies using a combination of external and genitalic features. The morphological terminology follows that of McAlpine (1981) . Absolute measurements for the body length are given in millimeters. Descriptions of the species were done in the following order: body length, head, thorax, wings, legs and abdomen. The type specimens of the new species described herein are deposited at the Institute of Entomology, Shenyang Normal University, China (IESNU).

### Nomenclature

This paper and the nomenclature it contains have been registered with ZooBank. The LSID number is:

urn:lsid:zoobank.org :pub:9CA1C0D0-773B-4123 - ABD2-49EC4E74DB1F

### Diagnosis of *Phaonia fuscicoxa-group*Ma, 2002

Scutellum and abdomen entirely black, anterior *acr* absent, posterior *dc* 4, *pra* longer than posterior notopleural seta, katepimeron bare, notopleuron with hairs; mid tibia with 1 row of *p,* hind tibia without apical *pv,* fore tarsi without sensitive hairs in ventral side.

### Key to the adult males of the *Phaonia fuscicoxa-group* from China

1. Vibrissal angle situated in front of frontal angle in profile *(Phaonia shanxiensis –* sub group) ………………………………………..2 — Vibrissal angle situated behind frontal angle in profile *(Phaonia fuscicoxa -* sub-sub-group) …………………….7
2. Palpus dark red-brown to dark brown; basal two segments of antenna and basal 1/4 of flagellomere 1 red-brown…………………….… *Phaonia mimoaureola*Ma, Ge and Li, 1992 — Palpus and antenna entirely black………..3
3. Femora entirely black………...…………..4 —Femora and tibiae yellow or brown-yellow……………………..5
4. Eyes covered with long ciliae, *fr* 9-11 pairs, genal height **∽** 1/5 of eye height; basal 1/3-1/4 of tibia black, basal half of mid femur with long and large *pv* row *,* hind tibia with 4 *av,* with 1 row of short medial *p* ……………….…… *Phaonia comihumera* Feng and Ma, 2002 — Eyes covered with short ciliae, *fr* 5 pairs, genal height about 1/3 of eye height; tibia entirely yellow, basal half of mid femur without *pv,* hind tibia with 2 *av,* without medial *p* ………….. *Phaonia liangshanica*Feng, 2004
5. Eyes covered with short ciliae, the upper and inner facets apparently enlarged; parafacial narrow, **∽** 1/2 of the width of flagellomere 1………………………………..…….. *Phaonia impigerata* Feng and Ma, 2002 — Eyes covered with long ciliae, the upper and inner facets not apparently enlarged; parafacial nearly equal to the width of flagellomere 1.………………………………6
6. Thorax and abdomen black; genal height 1/4 of eye height; Basicosta dark brown…………….…………………………...…… *Phaonia shanxiensis,*Xue and Cao, 1989 — Thorax and abdomen dark brown; genal height 1/3 of eye height; Basicosta yellow…………..…………………………..…………………… *Phaonia sichuanna*Feng, 2004
7. Basal half of cerci narrow, inner projecting in the distal part wide and long……………...8 — Basal half of cerci wide, inner projecting in the distal part narrow and short…………………………………………… *Phaonia kanoi*Shinonaga and Huang, 2007
8. Basal two segments of antenna and the basal part of flagellomere 1 yellow; palpus dark brown to brown-yellow……………………..9 — Antenna black to dark fuscous; palpus black to dark fuscous or only fuscous on distal part…..……………………………………..12
9. Hind femur with a complete row of *pv* ; eyes covered with short ciliae…………………...10 — Hind femur with *pv* only on basal 1/4-1/3; eyes covered with long ciliae………………11
10. Eyes almost bare, parafacial about equal to the width of antenna; flagellomere 1 ∽2.5x as long as wide, arista plumose, the longest hair equal to the width of antenna; thorax and abdomen with blue-gray pruinosity; *pra* ∽ 2.0 × as long as posterior notopleural seta; veins yellow; hind femur without *av* on basal half ; abdomen oviform, tergite 3-5 with black medial vitta, tergite 4 with incomplete posterior marginal seta row…………………………….………….. *Phaonia dianxiia*Li and Xue, 2001 — Eyes covered with short ciliae, parafacial **∽** 2/3 of the width of flagellomere 1; flagellomere 1 **∽** 3.5 × as long as wide, arista plumose, the longest hair **∽** 2.0 × as long as the width of flagellomere 1; thorax and abdomen with gray-brown pruinosity; *pra***∽** 1.5 × as long as posterior notopleural seta; veins fuscous; hind femur with complete *av* row; abdomen round, only tergite 3 with short black medial vitta on anterior 3/5, tergite 4 with complete posterior marginal seta row…... *……Phaonia debiliaureola*Xue and Cui, 1996
11. Middle femur without *pv;* middle tibia with 2 *p;* the upper and inner facets not enlarged; anterior genal margin with 1-2 rows of upcurved setae……………………………….. …………….. *Phaonia aureoloides* Hsue, 1984 —Middle femur with 3 *pv* on basal half, which thinner than the diameter of femur; middle tibia with 3 *p;* the upper and inner facets distinctly enlarged; anterior genal margin with only 1-2 upcurved setae………………...…….. *Phaonia subaureola* Feng and Ma, 2002
12. Palpus fuscous or only fuscous on distal part…………………………………………13 — Palpus entirely black…………………...14
13. Eyes covered with long ciliae, the longest hair of arista **∽** 2.0 × as wide as flagellomere *1, fr* 10-11 pairs, genal height **∽** 1/4 of eye height; palpus fuscous only on distal part; the inner vittae on scutellum reaching to scutoscutellar suture, anterior spiracle fuscous; basicosta fuscous; middle femur with a row of *pv* on basal half, hind tibia with 2 *ad* ……………………………………………...… *Phaonia chuanierrans*Xue *and Feng,* 1986 —Eyes covered with short ciliae, the longest hair of arista **∽** 1.5x as wide as flagellomere 1, *fr* 6 pairs, genal height **∽** 1/6 of eye height; palpus entirely fuscous; the inner vittae of scutellum not reaching to scutoscutellar suture, anterior spiracle light yellow; basicosta yellow; middle femur with 2 *pv* on basal part, hind tibia with 1 *ad* …………………………... … *.Phaonia debilifemoralis*Xue and Cui 1996
14. The upper and inner facets not apparently enlarged……………………………………15 — The upper and inner facets about 2.0× as large as the lower and outer ones…………….. … *.Phaonia macroomata*Xue and Yang, 1998
15. Legs entirely black………………….….16 — Legs at least femur and tibia brownish yellow…………………………………………17
16. Arista plumose, the longest hair equal to the width of flagellomere1; abdomen with large patches………….. *Phaonia hirtiorbitalis*Xue, Wang and Du, 2006 — Arista long plumose, the longest hair ∽1.5x as long as the width of flagellomere 1; abdomen without patch… *Phaonia nigrifuscicoxa*Xue, Wang and Du, 2006
17. Fore tibia with medial *p* ……………18 —Fore tibia without medial *p* ……………19
18. Hind femur without *pv;* abdomen without shifting patch, tergite 4-5 with bright black band in posterior margin…………………… *Phaonia subfuscicoxa* Xue and Rong, **sp. nov.** — Hind femur with 5-6 thick and short *pv* on basal 3/5; abdomen with shifting patches, tergite 4-5 without band in posterior margin…….. *Phaonia paradisia*Li and Xue, 2001
19. Anterior and posterior spiracles entirely light yellow; basal 2/3 of hind femur without distinct *pv* …………… *Phaonia tuberosurstyla*Deng and Feng, 1998 — Anterior and posterior spiracle entirely fuscous or dark fuscous; hind femur with *pv* on basal 2/3………………………………..20
20. *Pra* slightly shorter than posterior nopopleural seta; hind femur with complete and long *pv* row…………………………………... … *.Phaonia falsifuscicoxa*Fang and Fan, 1992 — *Pra* 2.0× as long as posterior nopopleural seta; hind femur with incomplete *pv* row, sometimes slightly weak or only 1………...21
21. Parafacial as long as width of flagellomere1; arista long plumose, the longest hair ∽2.0x as long as width of flagellomere1; basicosta black fuscous……………………… ………………… *Phaonia xixianga*Xue, 1996 — Parafacial narrow, not more than 3/5 to the width of flagellomere1; arista plumose or long plumose, the longest hair not more than 1.3x as long as flagellomere1; basicosta not black……………………………………….22
22. Eyes covered with dense and long ciliae, hind femur with complete *av* rows, with only 1 medial *pv;* hind tibia with 2 *ad* ………….. *Phaonia fuscicoxa*Emden, 1965 — Eyes covered with sparse and short ciliae, hind femur with incomplete *av* rows, with 3-4 *av* only on distal 1/4, *pv* setulae-like, slightly longer on basal part, only half width of hind femur; hind tibia with 1 *ad* ………… *Phaonia hypotuberosurstyla* Xue and Rong **, sp. nov.**

### Species in *Phaonia fuscicoxa-group* from China

1 *. Phaonia aureoloides Hsue,* 1984: 112

**Type material.** *Holotype.* Male, China: Qinghecheng, Benxi City, Liaoning Province, 14 June 1976, Coll. Wan-qi Xue. The type specimen is deposited at Liaoning Center for Disease Control and Prevention.

**Distribution.** China: Qinghecheng (124°23E, 41°46'N), Benxi City, Liaoning Province.

**Remarks.** This species resembles *P. subaureola* Feng and Ma, 2002, but the inner projection of the latter male’s cerci doesn’t extrude forward in lateral view, the distal margin distinctly sunken in posterior view.

2 *. Phaonia chuanierrans*Xue and Feng, 1986 : 413

**Type material.** *Holotype.* Male, China: Mt. Erlang, Luding County, Sichuan Province, 31 August 1983, Coll. Yan Feng. The type specimen is deposited at the IESNU.

**Distribution.** China: Mt. Er’ lang (102°24E, 29°91'N), Luding County, Sichuan Province.

**Remarks.** This species resembles *P. debilifemoralis*Xue and Cui, 1996 , but it differs from the latter in cerci of male with narrow and long inner projection, sustylus slightly long, the distal half enlarged.

3. *Phaonia comihumera* Feng and Ma, 2002: 330

**Type material.** *Holotype.* Male, China: Mt. Erlang, Ya’ an City, Sichuan Province, 2,680 m, 1 July 1988, Coll. Yan Feng. The type specimen is deposited at academy of military medical science of China.

**Distribution.** China: Mt. Er’ lang (102° 24E, 29° 91'N), Ya’ an City, Sichuan Province.

**Remarks.** This species resembles *P. liangshanica*Feng, 2004 , but the surstylus of male of the latter enlarge as triangle.

4. *Phaonia debiliaureola*Xue and Cui, 1996 : 366

**Type material.** *Holotype.* Male, China: Huaping, Guangxi Province, 700 m, 9 June 1993, Coll. Yong-shen Cui; *Paratypes. 2* males, same data as holotype. The type specimens are deposited at IESNU.

**Distribution.** China: Huaping (106°08E, 22°5'N), Guangxi Province.

**Remarks.** This species resembles *P. dianxiia*Li and Xue, 2001 , but differs from the latter in the inner projection of cerci of the male slightly short in posterior view, surstylus slightly long in lateral view.

5. *Phaonia debilifemoralis*Xue and Cui, 1996 : 368

**Type material.** *Holotype.* Male, China: Mt. Wuzhi, Hainan Province, 1,200 m, 4 May 1993, Coll. Yong-sheng Cu. The type specimen is deposited at IESNU.

**Distribution.** China: Mt. Wuzhi (109°07E, 18°09'N), Hainan Province.

**Remarks.** This species resembles *P. debilis* Stein, 1918, but can be distinguished from the latter by having nearly bare eyes; wider parafacialia; the longest aristal hair nearly 1.5x as long as the width of flagellomere 1; coxae yellow; hind femur *with pv.*

*6. Phaonia dianxiia* Li and Xue, 2001 :379

**Type material.** *Holotype.* Male, China: Pianma, Lushui, Yunnan Province, 1,800 m, 23 May 1992, Coll. Fu-hua Li. The type specimen is deposited at the IESNU.

**Distribution.** China: Pianma (98°38E, 26°10'N), Lushui, Yunnan Province.

**Remarks.** This species resembles *P. debiliaureola*Xue and Cui, 1996 , but it differs from the latter one in the inner projection of cerci of the male slightly long in posterior view, surstylus slightly short in lateral view.

7. *Phaonia falsifuscicoxa*Fang and Fan, 1992 : 1243

**Type material.** *Holotype.* Male, China: Huayanding, Mt. E’ mei, Sichuan Province, July 1981, Coll. Anxiao Deng; *Paratype.* 1 male, China: Yanzigou, Mt. Gongga, Sichuan Province, 2,300 m, 4 June 1983, Coll. Yuan-qing Chen. The type specimens are deposited at Shanghai Entomological Institute.

**Distribution.** China: Mt. E’ mei (103°48E, 29°59'N); Mt. Gongga (101°30'-102°15E, 29°20'-30°20'N), Sichuan Province.

**Remarks.** This species resembles *P. fuscicoxa* Emden, but it differs from the latter in the inner projection of cerci distinctly longer than the outer one, the cerci slightly wide in lateral view; the latter parafacial almost as wide as flagellomere 1; r4 _+_ 5 and m1+2 depart from each other in distal part; hind femur with only 1 *pv* in the middle, hind tibia with 3 *ad.*

8. *Phaonia fuscicoxa*Emden, 1965 : 278

**Type material.** *Holotype.* Male, NE. Burma: Kambaiti. The type specimen is deposited at Natural History Museum, London.

**Distribution.** Sichuan Province; Lushui: Pianma, Yuannan Provnce; NE. Burma: Kambaiti, Myitkyina (97°03E, 25°04'N); India; Nepal.

**Remarks.** This species resembles *P. falsifuscicoxa*Fang and Fan, 1992 , but the latter one parafacial -1/2 of the width of flagellomere 1; hind femur with a row *of pv,* hind tib-tibia with 2 *ad.*

9. *Phaonia hirtiorbitalis*Xue, Wang and Du, 2006 : 12

**Type material.** *Holotype,* Male, China: Mt. Paoma, 3,500 m, Sichuan Province, 14 July 2005, Coll. Liang Chang. *Paratype,* 1 male, same data as holotype. The type specimens are deposited at the IESNU.

**Distribution.** China: Mt. Paoma (101°E, 26°90'N), Sichuan Province.

**Remarks.** This species resembles *P. xixianga*Xue, 1996 , but it differs from the latter in having frontal setae short and dense, 14-16 pairs, those on upper half slightly longer than ciliae on eyes, those on lower half-2.0-3.0× as long as those on upper half, extend to both sides of anterior ocellus; without *ors;* arista plumose, the longest hair of arista about equal to the width of flagellomere 1; palpus dark brown on basal half and black on distal half, about equal in length to prementum; crossvein dm-cu straight; legs entirely black; mid tibia with 2 *p;* hind tibia with 2 *ad;* abdomen with versicolor patches.

10. *Phaonia impigerata* Feng and Ma, 2002: 327

**Type material.** *Holotype.* Male, China: Mt. Erlang, Ya’ an City, Sichuan Province, 2650 m, 1 July 1988, Coll. Yan Feng. *Paratype,* 1 male, same data as holotype. The type specimens are deposited at the Academy of Military Medical Science of China.

**Distribution.** China: Mt. Er’ lang (102°24E, 29°91'N), Ya’ an City, Sichuan Province.

**Remarks.** This species resembles *P. macroomata*Xue and Yang, 1998 , but the latter facets apparently enlarged, epi stoma and frontal angle situated on a vertical line; male cerci width longer than the length in posterior view, slightly wide in lateral view, surstylus slightly long.

11. *Phaonia kanoi*Shinonaga and Huang, 2007 :23

**Type material.** *Holotype.* Male, China: Tsuifeng, Taiwan Province, 20 May 1972, Coll. R.Kano. *Paratypes,* 4 males, 2 females, same data as holotype; 1 male, China: Mt. Ali, Jiayi County, Taiwan Province, 2,300 m, 3 June 1970, Coll. H. Kurahashi. The holotype is deposited in the collection of the National Science Museum, Tokyo, and all the paratypes are deposited at the National Museum of Natural Science, Taichung, Taiwan. **Distribution.** China: Tsuifeng (128°01'– 128°14'E, 45°38'–45°47'N), Mt. Ali (120°48'E, 23°31'N), Taiwan Province.

**Remarks.** This species resembles *P. shaanxiensis*Xue and Cao, 1989 , the differences between the two species in external feature are listed in the 7th item of the key.

12. *Phaonia liangshanica* Feng, 2004: 616

**Type material.** *Holotype.* Male, China: Meigu, Sichuan Province, 1600 m, 11 July 1975, Coll. Tao Ni. The type specimen is deposited at Shanghai Institute for Biological Sciences, CAS.

**Distribution.** China: Meigu (102°53'– 103°21'E, 28°02'–28°54'N), Sichuan Province.

**Remarks.** This species resembles *P. Shaanx-inensis*Xue and Cao, 1989 , but the latter male parafacial with *fr* on upper half, femur yellow; abdomen tergite 3 and 4 with black posterior band; cerci width not apparently longer than the length in posterior view.

13. *Phaonia macroomata*Xue and Yang, 1998 : 99

**Type material.** *Holotype.* Male, China: Mt. Changbai, Jilin Province, 28 July 1984, Coll. Ji-cheng Wang. *Paratypes.* 5 males, 1 female, same data as holotype. The type specimens are deposited at IESNU.

**Distribution.** China: Yanghugou (124°23'E, 41°33'N), Benxi City, Liaoning Province; Mt. Changbai (127°28'-128°16E, 41°42'– 42°25'N), Jilin Province.

**Remarks.** This species resembles *P. impigerata* Feng and Ma, 2002, but the latter genal height -1/3 of eye height, parafacial narrow, -1/2 of the width of flagellomere 1, epistoma slightly exceeds frontal angle; male cerci width shorter than length in posterior view, slightly narrow in lateral view, surstylus slightly short.

14. *Phaonia mimoaureola*Ma, Ge and Li, 1992 : 453

**Type material.** *Holotype.* Male, China: Mt. Jigong, Henan Province, 16 June 1983; 1 male, 1 female, 26 May 1983, Coll. Feng-xiang Ge. *Paratype.* 1 male, China: Shennongjia, Hubei Province, 31 May 1984, Coll. Yu Gao. The type specimens are deposited at Liaoning Center for Disease Control and Prevention.

**Distribution.** China: Mt. Jigong (114°01'– 114°06E, 31°46'–31°52'N), Henan Province; Shennongjia (109°56'–110°58E, 31°15'– 31°75'N), Hubei Province.

**Remarks.** This species differs from other species of *P. fuscicoxa-group* in having a palpus not black but dark red-brown; the inner projection of cerci in male extrudes forward in lateral view.

15. *Phaonia nigrifuscicoxa*Xue, Wang and Du, 2006 : 15

**Type material.** *Holotype,* Male, China: Hanmi, Mêdog, Tibet, 2,150-3,200 m, 9 August 2003, Coll. Ming-fu Wang. *Paratype,* 1 female, same data as holotype. The type specimens are deposited at IESNU.

**Distribution.** China: Hanmi (95°30'E, 29°20'N), Tibet.

**Remarks.** This species resembles *P. hirtiorbitalis,* but it differs from the latter in hav-having frons about equal width to the anterior ocellus; palpus black; mid tibia with 3 *p;* abdomen without versicolor patches; the inner injection of cerci in male shorter than the outer one.

16. *Phaonia par adisia*Li *and Xue,* 2001 : 380

**Type material.** *Holotype.* Male, China: Luquan, Yunnan Province, 3,500 m, 10 April 1987, Coll. Fu-hua Li. The type specimen is deposited at IESNU.

**Distribution.** China: Luquan (102°14'– 102°56E, 25°25'–26°22'N), Yunnan Province.

**Remarks.** This species resembles *P. fuscicoxa*Emden, 1965 , but it differs from the latter in the male eyes with sparse and short ciliae, frons broad, the longest hair of arista ∽2.0x than the width of flagellomere 1, postgena with light yellow hairs; lower and lateral ventral margins of scutellum with black hairs, anterior spiracle light yellow; coxae and trochanters yellow, fore tibia with 2 *p,* basal 3/5 of hind femur with 5-6 thick and short *pv.*

17. *Phaonia shaanxiensis*Xue and Cao, 1989 : 166

**Type material.** *Holotype* . Male, China: Mt. Taibai, Shanxi Province, 16 August 1982, Coll. Ru-feng Cao. The type specimen is deposited at IESNU.

**Distribution.** China: Mt. Taibai (107°22′– 107°51′E, 33°49′–34°05′N), Shanxi Province.

**Remarks.** This species resembles *P. comihumera* Feng *and* Ma, but the latter with basicosta yellow brown, femur black; the inner projection of cerci in male slightly short.

18. *Phaonia sichuanna*Feng, 2004 : 9

**Type material.** *Holotype* . Male, China: Nek of Mt. Er’ lang, Sichuan Province, 3,100 m, 21 August 1984, Coll. Yan Feng. The type specimen is deposited at Shanghai Institute for Biological Sciences, CAS.

**Distribution.** China: Nek of Mt. Er’ lang (102°24′E, 29°91′N), Sichuan Province.

**Remarks.** This species resembles *P. shaanxiensis*Xue and Cao, 1989 , but the latter body dark black, thorax with 4 black vittae; the outer projection of cerci in male distinctly short, the inner one long and broad.

19. *Phaonia subaureola* Feng *and* Ma, 2002: 331

**Type material.** *Holotype* . Male, China: Mt. Zhougong, Ya’ an City, Sichuan Province, 1,300 m, 17 June 1984, Coll. Yan Feng. The type specimen is deposited at the Academy of Military Medical Science of China.

**Distribution.** China: Mt. Zhougong (103°03′E, 30°N), Sichuan Province.

**Remarks.** This species resembles *P. aureoloides* Hsue, 1984, the differences between the two species in external feature are listed in the 11th item of the key.

**Figure 1.**
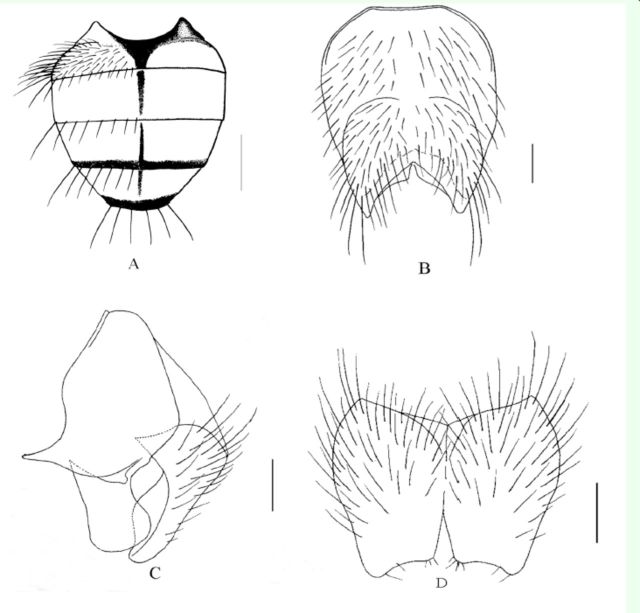
*Phaonia subfuscicoxa* Xue and Rong, **sp. nov.** A. Male, abdomen in dorsal view, scale = 1 mm; B. Male, sternite 5 in ventral view, scale = 0.2 mm; C. Male, cerci and surstyli in profile, scale = 0.1 mm; D. Male, cerci in posterior view, scale = 0.1 mm.

20. *Phaonia subfuscicoxa* Xue *and* Rong **sp. nov.** ( Fig. 1 A‒D)

**Holotype (Male)** . Body length 7.5 mm. *Head.* Eyes covered with long and dense brown ciliae, the upper facets not enlarged; frons narrow, equal to the width of anterior ocellus; frontal vitta black, situated on lower 1/3 of frons; *fr* 13‒14 pairs, including 6‒7 pairs thick and long on lower 2/5, 6 pairs thin and short on upper 3/5, shorter than ciliae on eyes; *ors* absent, ocellar seta long and large, as long as the lower *fr* , fronto-orbital plate and parafacial with brown-gray pruinosity, parafacial equal to or shorter than flagellomere 1; antenna black brown, flagellomere 1 -2.5 × as long as wide; arista long plumose, the longest hair ∽1.2-1.3* as wide as antenna; lunule red-brown; epistoma not projecting, vibrissal angle situated behind frontal angle; anterior margin of gena with 2-3 rows of upcurved setae, genal and occiput hairs black; gena height -1/4 of eye height; proboscis slender; prementum with dense gray pruinosity, -4.0-4.5 × as long as height; palpus black brown, thin and long, ∽1.2x as long as prementum; labella large, -1/2 as long as prementum.

*Thorax.* Black in background color, covered with sparse gray pruinosity; slightly shining; scutum with 4 black vittae, the inner vittae not reaching to scutoscutellar suture; *acr* 0+1; *dc* 2+4, *ial* 0+2, *pra* long and large, -2.0× as long as posterior notopleural seta; notopleuron with hairs; scutellum black, the flank and lower part bare, basisternum of prosternum, anepi sternum, katepimeron and meron all bare; katepisternal setae 1+2; spiracles brown.

*Wings.* Basal and anterior part slightly brown; basicosta brown; costal spine short and small; subcosta bend as a bow; r4 _+_ 5 and m1+2 veins slightly straight, radial node bare; the surrounding of r-m and dm-cu crossveins uncloud; calypteres light brown; halteres brownish yellow.

*Legs.* Femora and tibiae yellow, trochanters brown-yellow, coxae and tarsi black; fore tibia with 1 medial *p;* mid femur without distinct *av,* and with 1-2 *pv* on basal part, 1 *ad* and 3 *pd* on distal part; mid tibia with 3 *p,* present as a row; hind femur *av* row slightly complete but irregular, *pv* absent; hind tibia *av* 3, *ad* 3-4, the distal 1/5 with a long and *large pd,* without apical *pv;* the length of tarsi longer than tibiae, fore tarsus without long sensitive hairs; claws and pulvillus short, -3/5 of tarsomere 5.

*Abdomen.* Black, roundish in dorsal view, with blue-gray pruinosity, slightly shinning; without shifting patch; tergites with medium black vittae; tergite 3-5 with complete posterior marginal setae row; without distinct discal seta; tergite 1-2 with dense and short body hairs, tergite 3 with sparse and short body hairs; tergite 4-5 with slightly longer and sparse body hairs, the latter marginal without pruinosity, but with bright black band in posterior marginal; sternite 1 bare; sternites 2-4 with 1-2 pairs of strong setae on distal part. Sternite 5 lateral lobe short; distal part of cerci narrow in posterior view; surstyli wide apically in lateral view.

**Female** . Unknown.

**Type material.** *Holotype.* Male, China: Mt. Leigong (108°5'–108°24'E, 26°15'–26°32'N), Kaili, Guizhou Province, 2,100 m, 24 May 2011, Coll. Chun-tian Zhang; *Paratype.* 1 male, same data as holotype.

**Remarks.** This species belongs to the *Phaonia fuscicoxa-group,* it resembles *P. fuscicoxa*Emden, 1965 , but it differs from the latter in fore tibia with 1 medial *p,* hind femur without *pv,* mid femur with 1-2 *pv* on basal part, hind tibia with 3-4 *ad;* abdomen roundish in dorsal view, tergite 4-5 with bright black band in posterior margin.

**Etymology.** The specific name refers to its similarity to *P. fuscicoxa.*

**Distribution.** China: Guizhou Province.

21. *Phaonia tuberosurstyla*Deng and Feng, 1998 : 94

**Type material.** *Holotype.* Male, China: Zayü, Tibet, 15 June 1991, Coll. You-zhi Zhang. *Paratype.* 1 male, same data as holotype. The type specimens are deposited at Liaoning Center for Disease Control and Prevention.

**Distribution.** China: Zayü (96°52'–97°10'E, 28°34'–29°07'N), Tibet.

**Remarks.** This species resembles *P. xixianga*Xue, 1996 , but the latter has a black-brown basicosta; anterior and posterior spiracles brown; hind tibia with 2 *av* , mid femur with 4-6 *pv* on basal half; <$ the inner projection of cerci ∽2.0x as long as the outer one, the anterior margin of surstylus without tuber on basal part.

22. *Phaonia xixianga*Xue, 1996 : 1292

**Type material.** *Holotype.* Male, China: Mt. E’mei, Sichuan Province, 18 August 1957, Coll. Fu-xing Zhu. The type specimen is deposited at the Chinese Academy of Sciences.

**Distribution.** China: Xixiangchi, Mt. E’mei (103°48E, 29°59'N), Sichuan Province.

**Remarks.** This species resembles *P. tuberosur styla*Deng and Feng, 1998 , but the latter basicosta yellow; anterior and posterior spiracles light yellow-white; hind tibia with 1 *av* , mid femur with only 2 thick setae on ventral; the inner projection of cerci in male slightly longer than the outer one, the anterior margin of surstylus with a tuber on basal part.

**Figure 2.**
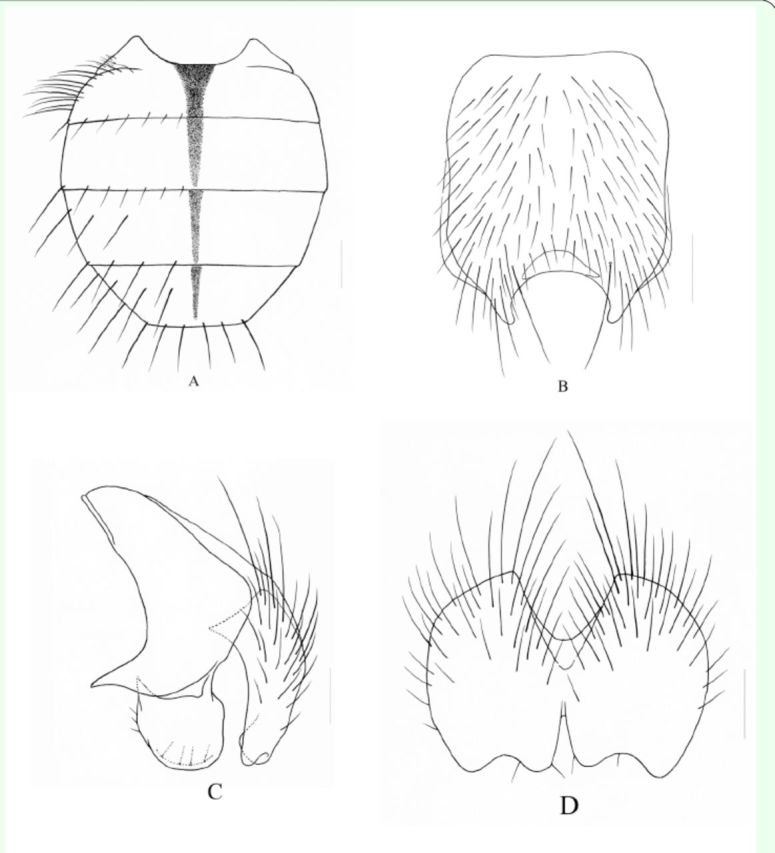
*Phaonia hypotuberosurstyla* Xue and Rong, **sp. nov.** A. Male, abdomen in dorsal view, scale =0.5 mm; B. Male, sternite 5 in ventral view, scale = 0.2 mm; C. Male, cerci and surstyli in profile, scale = 0.1 mm; D. Male, cerci in posterior view, scale = 0.1 mm.

23. *Phaonia hypotuberosurstyla* Xue and Rong, **sp. nov.** ( Fig. 2A -C)

**Holotype (Male)** . Body length 7.4-7.6 mm. *Head.* Eyes covered with short and sparse ciliae, the upper inner facets not enlarged; frons narrower than the width of anterior ocellus; frontal vitta black, upper half of fronto-orbital plate neighbouring, *fr* 5-6 pairs, situated on lower 2/5 of parafacial, *ors* absent; fronto-orbital plate, parafacial and gena with light gray pruinosity, parafacial —1/2—3/5 of the width of flagellomere 1, lunule yellow; scape, pedicel and basal of flagellomere 1 red-brown, flagellomere 1 ∽3.3x as long as wide, ∽2.5x as long as pedicel; arista long plumose, the longest hairs at least 1.3x than the width of flagellomere 1; gena height -1/5 of eye height; epistoma not projecting; anterior margin of gena with a row of upcurved setae, genal and occiput hairs all black; palpus black-brown, about as long as prementum, prementum covered with pruinosity, ∽2.5-2.7x as long as height; posterior margin of labellum with a big tooth in the middle.

*Thorax.* Black in background color, covered with brown-gray dense pruinosity; scutum with 4 black vittae, the inner pair reaching to scutoscutellar suture; scutellum black, lower lateral scutellar with 2-3 black hairs; *acr* 0+1, *dc* 2+4, *pra* ∽2.0x as long as posterior notopleural seta; notopleuron with 1-2 setae, sometimes bare on one side; katepisternal setae 1+2; anterior spiracles brown, posterior spiracles dark brown; basisternum of prosternum, anepi sternum, katepimeron and meron all bare.

*Wings.* With brown, and not hyaline; basicosta yellow; radial node bare; m-m vein slightly straight; calypteres and halteres brown-yellow.

*Legs.* Entirely yellow except dark tarsi; fore tarsus without long sensitive hairs, fore tibia without medial *p;* mid femur without *av,* and with 2-3 *pv* on basal 1/3; mid tibia with 2 *p;* hind femur with incomplete *av* row, and only with 3-4 *av* on distal 1/4, *pv* setulae-like, slightly longer on basal part, only 1/2 as long as the diameter of hind femur; hind tibia *av* 3, *ad* 1, *pd* 1, without apical *pv* .

*Abdomen.* Dark black, roundish in dorsal view, with gray to brown-gray pruinosity, body hairs dense and short, tergites with medial black vittae which not shifting, the width of vitta equal to its diameter, without shifting patches; posterior marginal seta row complete, discal seta 3–4 pairs; tergite 3 without medial marginal seta, lateral posterior marginal with 4‒5 setae; sternite 1 bare; sternite 2‒5 each with a pair of strong setae on distal part; the inner projection of cerci apparently longer than the outer one, surstyli wide and short in lateral view.

**Female** . Unknown.

**Type material.** *Holotype.* Male, China: Mt. Gaoligong (98°34'–98°50'E, 24°56'–26°09'N), Yunnan Province, 2,500 m, 19 July 2008, Coll. Shu-chong Bai. *Paratypes.* 2 males, same data as holotype, Coll. Wen-xiu Dong.

**Remarks.** This species belongs to the *Phaonia fuscicoxa-group,* and resembles *P. tuberosurstyla*Deng and Feng, 1998 , but it differs from the latter in having a narrow frons, narrower than the width of anterior ocellus; arista long plumose, the longest hair at least 1.3x the width of flagellomere 1; anterior spiracles brown, posterior spiracles dark brown; hind tibia with 3 *av;* abdomen roundish in dorsal view, sternite 2-5 each with a pair of strong seta on distal part; cerci slightly wide in dorsal view, the inner projection longer than the outer one, surstyli wide and short in lateral view.

**Etymology** . The specific name refers to its similarity to *P. tuberosurstyla,* the Greek word “hypo-” refers to likeness.

**Distribution.** China: Yunnan Province.

**Diagnosis of** *Phaonia barkama-group* **Ma, 1998**

Without *ors,* basisternum of prosternum bare, katepimeron with hairs present or absent, notopleuron with hairs present, posterior *dc* 4; mid tibia with 2 rows of *p* (namely *p* and *pv);* abdomen without yellow parts and not hyaline.

### Key to the adult males of *Phaonia barkama* -group from China

1. Legs yellow except for black tarsi………… *………Phaonia daliensis* Xue and Du, **sp. nov.** — Legs entirely black, sometimes only the distal part of femur and basal part of tibia red-brown………………………………………..2
2. Katepimeron bare…………………………3 — Katepimeron with hairs…………………..4
3. Anterior *acr* absent; fore tibia without medial *p* …………………………………………. ……... *Phaonia maoershanensis* Xue, **sp. nov.** — Anterior *acr* 1; fore tibia with 1 medial *p* …… *Phaonia quadratilamella* Xue, **sp. nov.**
4. Hind tibia without additional *pd* ………….5 — Hind tibia with additional *pd* …………….6
5. Fore tibia with 2 *p* ; hind tibia with 4‒5 *av* , 3 *ad* ; abdomen with medium narrow black vittae only on tergite 3 and 4…………………….………... *Phaonia jiaodingshanica*Feng, 2004 — Fore tibia with 1 *p* ; hind tibia with 3 *av* , 2 *ad* ; abdomen with medium narrow black vittae on each tergite… *Phaonia cineripollinosa*Xue, Tong and Wang, 2008
6. *fr* only on the lower 3/5 of fronto-orbital plate, the upper part bare; m-m crossvein clouded; the upper inner facets enlarged………….. *Phaonia barkama*Xue, 1998 — *fr* row complete; m-m crossvein unclouded; the upper inner facets not enlarged……...7
7. Fore tibia without *p* ………………………... ……… *Phaonia caudilata*Fang and Fan, 1992 — Fore tibia with 1–2 medial *p* ……………..8
8. Abdomen without shifting patch on the lateral of tergite………………………………...9 — Abdomen with shifting patches on the lateral of tergite……………………………….10
9. Arista plumose, the longest hair about equal to width of antenna; the distal part of femora and the basal part of tibiae with red-brown, hind femur with thin and short *pv* ………………………...…………………… ….. *Phaonia yingjingensis* Feng and Ma, 2002 — Arista long plumose, the longest hair ∽2.0x as long as the width of antenna; legs entirely black, hind femur without *pv* …… *Phaonia caesiipollinosa* Xue and Rong, **sp. nov.**
10. Frons wide, ∽2.0x the width of anterior ocellus, fronto-orbital plate without pruinosity; abdomen without band in posterior margin on tergite… *..Phaonia nigriorbitalis*Xue, 1998 — Frons narrow, about equal to the width of anterior ocellus, fronto-orbital plate with pruinosity; abdomen with gray or black band in posterior margin at least on tergite 3-5…………………………………………11
11. Fronto-orbital plate and parafacial with brown pruinosity; gena with 1 row of up-curved subvibrissal setula; mid femur with complete *pv* row; hind tibia with 3 *av* …………... *Phaonia nigribasalis*Xue, 1996 — Fronto-orbital plate and parafacial with gray pruinosity; gena with 2 rows of upcurved subvibrissal setula; mid femur with 2-3 *pv* only on basal 1/3; hind tibia with 2 *av* …………………… *Phaonia aureipollinosa*Xue and Wang, 1986

### Species of *Phaonia barkama-group* from China

*1.Phaonia aureipollinosa* Xue and Wang, 1986 : 321

**Type material.** *Holotype.* Male, China: Guan-ling, Shanxi Province, 9 July 1983, Coll. Ming-fu Wang. *Paratype.* 1 male, China: Weiyuan, Gansu Province, July 1982, Coll.

Jing-wei Wu. The type specimens are deposited at IESNU.

**Distribution.** China: Guangling (113°51– 114°24'E, 39°35'–39°55'N), Shanxi Province; Weiyuan (104°22'E, 35°14'N), Gansu Province.

**Remarks.** This species resembles *P. nigribasalis*Xue, 1996 , but the latter with only 1 row of upcurved subvibrissal setula on gena; mid femur with complete *pv* row; abdomen with medium black vittae on each tergite, and with shifting patches on both sides.

2. *Phaonia barkama*Xue, 1998 : 1190

**Type material.** *Holotype.* Male, China: Maerkang, Aba, Sichuan Province, 3,280 m, 2 July 1961, Coll. Suo-fu Li. The type specimen is deposited at Chinese Academy of Sciences.

**Distribution.** China: Maerkang (101°17'– 102°41'E, 30°35'–32°24'N), Aba, Sichuan Province.

**Remarks.** This species resembles *P. nigribasalis*Xue, 1996 . But the latter with upper inner facets not enlarged; with complete *fr* row, the upper ones thin and small.

**Figure 3.**
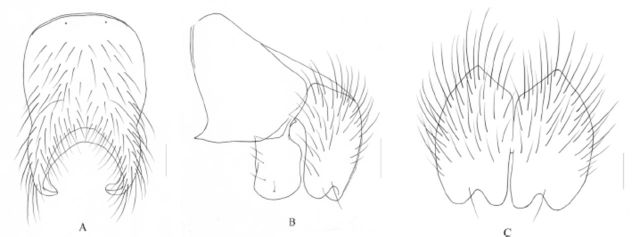
*Phaonia caesiipollinosa* Xue and Rong, **sp. nov.** A. Male, sternite 5 in ventral view, scale = 0.2 mm; B. Male, cerci and surstyli in profile, scale = 0.1 mm; C. Male, cerci in posterior view, scale = 0.1 mm.

3. *Phaonia caesiipollinosa* Xue and Rong, **sp. nov.** ( Fig. 3A -C)

**Holotype (Male)** . Body length 7.4-7.6 mm. *Head.* Eyes covered with light yellow and slightly sparse long ciliae; frons ∽1.0-1.2x as wide as anterior ocellus; frontal vitta black, the narrowest part presents as a line; ocellar seta long and large, as long as the longest *fr* ; *fr* 11-13 pairs, reaching to both sides of anterior ocellus; upper 4-5 pairs distinctly short and about equal to length of ciliae on eyes; *ors* absent; fronto-orbital plate and parafacial with light gray pruinosity, parafacial ∽1.2x than width of flagellomere 1; antennae black, flagellomere 1 ∽2.5-2.8x as long as wide; arista long plumose, the longest hair -2.0× than width of antenna; lunule brown-yellow; Vibrissal angle situated behind frontal angle; genal height -1/4 of eye height; anterior margin of gena with 2 rows of irregular upcurved setae, genal, epicephalon, and occiput hairs black; prementum with pruinosity, -2.5-3.0× as long as height; palpus black, about equal to length of prementum.

*Thorax.* Black in background color, covered with sparse light gray pruinosity and slightly shining; scutum with brown-gray pruinosity on posterior half, scutum with 4 black vittae, the inner vittae reaching to the middle of scutoscutellar suture, and about half of the length of medium light pruinosity vitta which in front of scutum; *acr* 0+1; *dc* 2+4, *ial* 0+2, *pra* -2.0-2.3 × as long as posterior notopleural seta; the flank and lower part of scutellum bare, notopleuron and katepimeron covered with most black hairs; basisternum of prosternum, anepisteron and meron all bare; katepisternal setae 1+2; spiracles dark brown, slightly large.

*Wings* . Slightly brown on basal part, veins all brown; basicosta black; costal spine short and small, ≈3/5 as long as r-m crossvein; subcosta bend as a bow; the surrounding of r-m and dm-cu crossveins uncloud; radial node bare; dm-cu crossvein slightly bend as an “S”, r _4+5_ and m _1+2_ veins straight, slightly paralleled; calypteres brown, lower calypter ligulate; halteres light yellow, basal part brown.

*Legs* . Entirely black; fore tibia with 1–2 medial *pv* ; mid femur with hair-like *av* on basal 1/3, with 3 *pd* near apical part, 3–4 thick *pv* on basal 2/5; mid tibia with 3 *p* , 1–2 *pv* ; hind femur *av* row complete, sparse and short, *pv* absent; hind tibia with 3 *av* , 3 *ad* , 1 long and large *pd* on apical 1/4, 1 small *pd* on subbasal part, and with a row of short setae on posterior part, the longest seta about as long as the diameter of hind tibia, ≈4‒5, without apical *pv* ; the length of tarsi longer than tibiae; claws and pulvillus equal length; fore legs claws and pulvillus about equal to length of tarsomere 5; mid and hind legs claws and pulvillus ≈3/5 of length of tarsomere 5.

*Abdomen* . Black in ground color, oviform in dorsal view, with homogeneous light gray to blue‒gray sparse pruinosity, without shifting patch; tergite 2‒5 with medium black narrow vitta, without band in posterior marginal; tergite 3-5 with complete setae row in posterior marginal, body hairs dense, slightly short in the middle and gradually longer on both sides, tergite 5 covered with sparse and shining pruinosity on posterior; sternite 1 with 1-2 pairs of thin hairs, sternite 2 with 2 pairs of long setae. Sternite 3 and 4 with sparse and short body hairs, with a pair of thick setae on posterior part; Sternite 5 lateral lobe short and small.

**Female** . Unknown.

**Type material.** *Holotype.* Male, China: Da-tulugou (102°82'E, 36°67'N), Yongdeng, Gansu Province, 2,300-2,350 m, 20 July 2009, Coll. Zhe Zhao. *Paratypes.* 2 males, same data as holotype, Coll. Shuai Wang.

**Remarks.** This species belongs to the *Phaonia barkama-group,* and resembles *P. aureipollinosa*Xue *and* Wang, 1986 , but it differs from the latter in eyes covered with light yellow and slightly sparse long ciliae; calypter brown; hind tibia with 3 *av,* 3 *ad,* and with a row of short setae on posterior part, longest seta about equal to diameter of hind tibia, ∽5-6; abdomen with homogeneous light gray to blue-gray sparse pruinosity, tergites without shifting patch and band in posterior marginal; sternite 1 with 1-2 pairs of thin hairs; cercus distinctly sunk on distal part, the outer projecting shorter than inner one, surstylus slightly long and rectangle.

**Etymology** . The specific name refers to its similarity to *P. aureipollinosa*Xue *and* Wang, 1986 , but the new species has blue-gray pruinosity on abdomen. The Latin word “caesi” refers to blue-gray.

**Distribution.** China: Gansu Province.

4. *Phaonia caudilata*Fang and Fan, 1992 : 1241

**Type material.** *Holotype.* Male, China: Mt. Meili, Deqin, Yunnan Province, 22 July 1982, Coll. Huai-cheng Chai. The type specimen is deposited at The Chinese Academy of Sciences, Shanghai Institute of Insects.

**Distribution.** China: Mt. Meili (98°83'E, 28°47'N), Deqin, Yunnan Province.

**Remarks.** This species resembles *P. aureipollinosa*Xue and Wang, 1986 , but the latter fore tibia with 1-2 medial *p;* arista long plumose, the longest hairs ∽2.0x as long as the width of flagellomere 1; anterior and posterior spiracle dark brown; Abdomen with brassy yellow dense pruinosity.

5. *Phaonia cineripollinosa*Xue, Tong and Wang, 2008 : 167

**Type material.** *Holotype.* Male, China: Hanmi, Metok, Tibet, 2,150-3,200 m, 9 August 2003, Coll. Ming-fu Wang. *Paratypes. 2* females, same data as holotype. The type specimens are deposited at IESNU.

**Distribution.** China: Metok (95°33E, 29°33'N), Tibet.

**Remarks.** This species resembles *P. aureipollinosa*Xue and Wang, 1986 , but it differs from the latter in genal height ∽2/5 of eye height; thorax with sparse and gray pruinosity; hind femur with obvious *av* row only in distal half, without *pv;* hind tibia with 3 *av, 2 ad,* and with 1 *pd* on distal 1/4, in subbasal without *pd,* and without apical *pv;* abdomen with sparse gray pruinosity, tergites with black narrow medium vittae, without band in posterior margin and shining patch.

**Figure 4.**
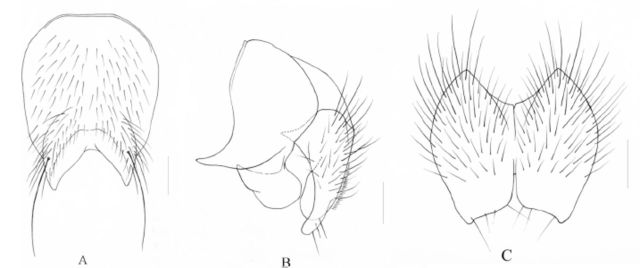
*Phaonia daliensis* Xue and Du, sp. nov. A. Sternite 5 in ventral view, scale = 0.2 mm; B. Male, cerci and surstyli in profile, scale = 0.1 mm; C. Male, cerci in posterior view, scale = 0.1 mm.

6. *Phaonia daliensis* Xue and Du, **sp. nov.** ( Fig. 4A -C)

**Holotype (Male)** . Body length 7.4-7.6 mm. *Head.* Eyes covered with brown and dense long ciliae; frons narrow, shorter than width of anterior ocellus; fronto-orbital plate adjoin in the middle; *fr* 11-12 pairs, up to both sides of anterior ocellus, upper 5-6 pairs thin and short and about equal to length of ciliae on eyes; *ors* absent; fronto-orbital plate and parafacial and gena with gray pruinosity, parafacial about equal to the width of flagellomere 1; lunule dark brown; antenna most black, pedicel with brown in distal part; arista long plumose, the longest hair ∽1.8x than width of antenna; flagellomere 1 ∽2.8x as long as wide; facial carina slightly lower; epistoma not projecting to frontal angle; genal height ∽2/9 of eye height; anterior margin of gena with 2 rows of upcurved setae, genal and occiput hairs entirely black; prementum black brown, with gray pruinosity, ∽2.8x as long as height; palpus black, longer than prementum; labellar large, -1/2 of length of palpus.

*Thorax.* Black in background color, covered with gray pruinosity; scutum with 4 black-brown vittae, the inner vitta not reaching to scutoscutellar suture; with 1 small seta on left front of the first *dc, acr* 0+1; *dc* 2+4, *ial 0+2, pra* long and large, -2.0× as long as posterior notopleural seta; scutellum black, the flank and lower part bare; notopleuron with hairs, basisternum of prosternum, proepi sternum concavity, anepisteron, katepimeron and meron all bare; anterior spiracle brown, posterior spiracle fuscous; katepi sternal setae 1+2.

*Wings.* With brown color; basicosta and subcostal sclerite brown-yellow; costal spine short and small; radial node bare; r4 _+_ 5 and m1+2 veins straight, the surrounding of r-m and dm-cu crossveins uncloud; calypteres brown-yellow, lower calypter extrude; halteres brown in basal, brown-yellow on distal part.

*Legs.* Yellow except for black tarsi; fore tibia with 1 medial *p;* mid femur with setalike *av* row, 3 *pv* on basal half, 1 *ad* near distal part and 4 *pd* arrange as a row; mid tibia without *ad,* 3 *p,* 2-3 *pv;* hind femur *av* row complete, slightly sparse, *pv* absent; hind tibia with 3 *av* and 2 long large *ad* in the middle, 1 long and large *pd* on distal 1/5, subbasal part without additional *pd* (only a short one on one side of 1 specimen), without apical *pv;* the length of tarsi longer than tibia; fore leg claws and pulvillus -4/5 as long as tarsomere 5.

*Abdomen.* Brilliant black, with sparse gray pruinosity, oviform in dorsal view, without shifting patch; tergite with medium black vittae, narrower on posterior part, and indistinct; tergite 4 and 5 with complete posterior marginal setae row and 2-3 pairs of discal setae, tergite 3 with posterior marginal setae only 2-3 long and large on flank; sternite 1 bare, sternite 5 lateral lobe with a pair of long seta; the inner projecting of cercus extrudes forward.

**Female** . Unknown.

**Type material.** *Holotype:* Male, China: Cangshan (110°17'E, 25°64'N), Dali, Yunnan Province, 2,400-2,500 m, 17 May 2012, Coll. Xiang Zhang. *Paratypes.* 2 males, same data as holotype, Coll. Hua Rong.

**Remarks.** This species differs from the other species in the *Phaonia barkama-group* in legs yellow except for black tarsi; male terminalia resembles *P. nigribasalis*Xue, 1998 , but anterior margin of gena with 2 rows of upcurved setae, katepimeron bare; basicosta brown-yellow; mid femur with incomplete *pv,* only 3 on the basal half; abdomen tergite without band in posterior margin.

**Etymology** . The specific name refers to its type locality.

**Distribution.** China: Yunnan Province.

**Figure 5.**
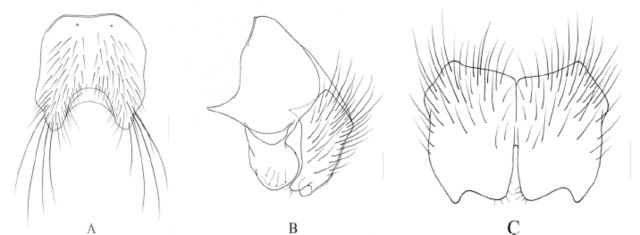
*Phaonia quadratilamella* Xue, **sp. nov.** A. Male, sternite 5 in ventral view, scale = 0.2 mm; B. Male, cerci and surstyli in profile, scale=0.1 mm; C. Male, cerci in posterior view, scale = 0.1 mm.

7. *Phaonia quadratilamella* Xue, **sp. nov.** ( Fig. 5A -C)

**Holotype (Male)** . Body length 6.0-6.2 mm. *Head.* Eyes covered with slightly sparse long ciliae; frons ∽1.5x than width of anterior ocellus; frontal vitta black, -1/2 of width of one side frons; *fr* 11-12 pairs, upper 5-6 pairs distinctly short and shorter than length of ciliae on eyes; *ors* absent, fronto-orbital plate, parafacial and gena with light gray pruinosity, parafacial about equal to width of antenna; lunule dark brown; antenna black, flagellomere 1 -2.5 × as long as wide; arista long plumose, the longest hairs ∽1.3x than width of antenna; epistoma behind frontal angle; anterior margin of gena with 1 row of upcurved setae, genal height -1/3 of eye height; genal, postgena and occiput hairs all black; prementum with pruinosity, ∽2.5x as long as height; palpus black, ∽1.2x than length of prementum.

*Thorax.* Black in background color, covered with sparse pruinosity, slightly shifting; with light narrow pruinosity strip along *dc* row, others black; *acr* 0+1; *dc* 2+4, *ial* 0+2, *pra* ∽1.5x as long as posterior notopleural seta; notopleuron with short hairs, scutellum the flank and lower part bare; basisternum of prosternum, proepi sternum concavity, anepisteron, katepimeron and meron all bare; upper proepi sternal seta 1, katepi sternal seta 1+2.

*Wings.* Brown in background color, veins fuscous; basicosta dark brown; costal spine short and small; subcosta bend as a bow; radial node bare; r4 _+_ 5 and m1+2 veins straight, slightly depart from each other on distal part; the surrounding of r-m and dm-cu crossveins uncloud; calypteres yellow, lower calypter extrudes as ligule; halteres brown-yellow.

*Legs.* Entirely black; fore tibia without medial *p;* mid femur without *av* , with 5-6 *pv* on basal 3/5; mid tibia with 3 *p, 2 pv;* hind femur *av* irregular, with thin setalike *pv* on distal 2/3, middle 4-5 slightly long, longer than diameter of hind femur; hind tibia with 3 *av* , 2 *ad,* 1 long and large *pd* near distal part, and 1-2 short *pd* on basal part, without apical *pv;* the length of tarsi longer than tibiae, claws and pulvillus equal length, ∽2/3 than length of tarsomere 5.

*Abdomen.* Black in background color, oviorm in dorsal view, with light gray to blue-gray dense pruinosity, without shifting patch, pruinosity slightly sparse in posterior, slightly shifting; tergite 2-5 with medium black narrow vittae; tergite 4 and 5 with complete pos-posterior marginal setae row and 3–4 pairs of discal setae; tergite 3 with short posterior marginal setae in the middle, like body hairs; sternite 1 bare, sternite 5 quadrate, lateral lobe with 3–4 long setae; the inner projecting of cercus wide and large.

**Female** . Unknown.

**Type material.** *Holotype* . Male, China: Bai-ma Snow Mountain (98°57′–99°25′E, 27°24′– 28°36′N), Yunnan Province. 4,250 m, 26 July 2008, Coll. Shuai Wang. *Paratype* .1 male, same data as holotype, Coll. Xue-shu Zhang.

**Remarks.** This new species belongs to the *Phaonia barkama* -group, male terminalia resembles *P. caudilata*Fang and Fan, 1992 , it differs from the latter in mid tibia with 3 *p* , 2 *pv* ; hind femur with complete *av* row, with *pv* ; cercus not narrow in distal half, and slightly narrow in profile view.

**Etymology** . The specific name refers to its quadrate sternite 5. The Latin word “quad-ratus” means quadrate, and “lamellate” means sheet.

**Distribution.** China: Yunnan Province.

8. *Phaonia jiaodingshanica*Feng, 2004 : 10

**Type material.** *Holotype* . Male, China: Mt. Jiaoding, Hanyuan, Sichuan Province, 3,550 m, 10 July 1988, Coll. Yan Feng. The type specimen is deposited at Shanghai Institute for Biological Sciences, CAS.

**Distribution.** China: Mt. Jiaoding (102°88 E, 29°37′N), Hanyuan, Sichuan Province.

**Remarks.** This species resembles *P. nigribasalis*Xue, 1996 , it differs from the latter in having a vibrissa not bending as an “N”; hind tibia without 1 *pd* on subbasal part; abdomen without patch; male surstylus not bend backward in profile view, and round oval on distal part.

**Figure 6.**
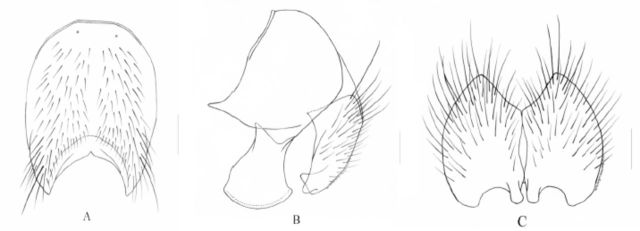
*Phaonia maoershanensis* Xue, **sp. nov.** A. Male, sternite 5 in ventral view, scale = 0.2 mm; B. Male, cerci and surstyli in profile, scale = 0.1 mm; C. Male, cerci in posterior view, scale = 0.1 mm.

9. *Phaonia maoershanensis* Xue, **sp. nov.** ( Fig. 6A -C)

**Holotype (Male)** . Body length 6.5-6.8 mm. *Head.* Eyes covered with light brown dense ciliae; frons narrow, ∽1.0-1.2* than width of anterior ocellus; fronto-orbital plate adjoin in the middle, *fr* 13 pairs, extend to both sides of anterior ocellus, the upper 6 pairs thin and small, about equal to length of ciliae on eyes; *ors* absent, fronto-orbital plate, parafacial and gena with gray pruinosity, parafacial ∽1.2x than width of antenna; lunule brown-yellow; antenna black, arista long plumose, most hairs ∽1.5x than width of antenna; flagellomere 1 ∽3.0x as long as wide; facial carina lower, epistoma not projecting; genal height ∽2/7 of eye height, genal, postgena hairs entirely black; anterior margin of gena with 1 row of upcurved setae; prementum black brown, with gray pruinosity, ∽3.0x as long as height; palpus black, longer than prementum; labellum medium, slightly longer than height of prementum.

*Thorax.* Black in background color, covered with gray pruinosity, with 4 indistinct black brown vittae; with 1 small seta in left front of the 1st *dc; acr* 0+1; *dc* 2+4, *ial* 0+2, *pra* long and large, ∽2.0x as long as posterior notopleural seta; scutellum black, the flank and ventral part bare; notopleuron with hairs; basisternum of prosternum, proepi sternum concavity, anepisteron, katepimeron and meron all bare; spiracles brown; katepisternal setae 1+2.

*Wings.* With brown, basicosta black; subcostal sclerite brown-fuscous, costal spine short and small; radial node bare; r4 _+_ 5 and m1+2 veins straight; the surrounding of r-m and dm-cu crossveins uncloud; calypteres light yellow, lower calypter extrudes; halteres brown-yellow on basal part, yellow on distal part.

*Legs.* Entirely black; fore tibia with 1 sub-medial *p;* mid femur without *av,* with 3-4 long and large *pv* on basal half, 1 *a* and 3 *pd* near distal part; mid tibia without *ad,* with 3 *p,* 1 *pv;* hind femur *av* row distinct, setalike on basal half, long and large on distal part, irregular, and without *pv;* hind tibia with 3 *av* in the middle, 3-4 *ad,* 1 long and large *pd* on distal 1/4, without *p* and *pv* ; the length of tarsi longer than tibiae; claws and pulvillus of fore legs long and large, equal to length of tarsomere 5; claw and pulvillus of mid and hind legs ∽4/5 of length of tarsomere 5.

*Abdomen.* Shifting black in background color, with gray pruinosity, oviform in dorsal view, without shifting patch; tergite 3, tergite 4 and 5 with medium black vitta on anterior half; tergite 3-5 with complete posterior marginal setae row, tergite 4 and 5 with about 4 pairs of discal seta row, break off in the middle; sternite 1 bare, surstylus axelike in profile view, the inner projecting of cercus slightly narrow.

**Female** . Unknown.

**Type material.** *Holotype.* Male, China: Mt. Maoer (110°19'–110°31'E, 25°44'–25°58'N), Guangxi Province, 2,000-2,200 m, 10 June 2012, Coll. Xiang Zhang. *Paratype.* 1 male, data same as holotype, Coll. Hua Rong.

**Remarks.** This new species belongs to *Phaonia barkama-group,* resembles *P. quadratilamella* Xue, **sp. nov.** , it differs from the latter in having the fronto-orbital plate adjoin in the middle, lunule brown-yellow, without anterior *acr,* fore tibia without 1 sub-medial *p;* surstylus axelike in profile view, the inner proj ecting of cercus slightly narrow.

**Etymology** . The specific name refers to its type locality.

**Distribution.** China: Guangxi Province.

10. *Phaonia nigriorbitalis*Xue, 1996 : 1249

**Type material.** *Holotype.* Male, China: Jiangda County, Changdu Area, Tibet, 3,400 m, 23 July 1976, Coll. Xue-zhong Zhang. The type specimen is deposited at Chinese Academy of Sciences.

**Distribution.** China: Jiangda County (97° 15'– 98°53'E, 31°00'–32°36'N), Changdu, Tibet.

**Remarks.** This species resembles *P. yingjinggensis* Feng *and* Ma, 2002, but the latter fronto-orbital plate with silvery-gray pruinosity; costal spine degenerates; abdomen tergite with medium black vitta, without shifting patch.

11. *Phaonia nigribasalis*Xue, 1996 : 1245

**Type material.** *Holotype.* Male, China: Sangdui, Daocheng County, Sichuan Province, 3950 m, 8 June 1982, Coll. Shu-yong Wang. The type specimen is deposited at Chinese Academy of Sciences.

**Distribution.** China: Sangdui (110° 11E, 29°19'N), Daocheng County, Sichuan Province.

**Remarks.** This species resembles *P. aureipollinosa*Xue *and* Wang, 1986 . But the latter gena with 2 rows of upcurved subvibrissal setula; mid femur with 2-3 *pv* only on basal 1/3; abdomen tergite with indistinct medium vitta.

12. *Phaonia yingjinggensis* Feng *and* Ma, 2002: 43

**Type material.** *Holotype.* Male, China: Paocaowan, Yingjing, Sichuan Province, 2,400 m, 1 June 1990, Coll. Yan Feng. *Paratypes. 2* males, same data as holotype; 1 male, China: Mt. Tuanbao, Hanyuan, Sichuan Province, 2,400 m, 10 July 1988, Coll. Li-fu Feng. The type specimens are deposited at Liaoning Center for Disease Control and Prevention.

**Distribution.** China: Paocaowan (102°52′–102°55′E, 29°30′–29°40′N), Yingjng; Hanyuan (102°16′–103°00′E, 29°05′– 29°43′N), Sichuan Province.

**Remarks.** This species resembles *P. nigriorbitalis*Xue, 1996 , but the latter has the epistoma situated behind anterior margin of frons; with distinct costal spine; fronto-orbital plate black, without pruinosity; abdomen tergite with narrow medium vitta, and with distinct shifting patches on both sides.

## Abbreviations

*fr*: frontal setae
*ors*: orbital setae
*acr*: acrostichal setae
*dc*: dorsocentral setae
*pra*: prealar setae
*av*: anteroventral setae
*ad*: anterodorsal setae
*pd*: posterodorsal setae
*pv*: posteroventral setae
*d*: dorsal setae
*p*: posterior setae
*Coll*: Collected
*IESNU*: Institute of Entomology, Shenyang Normal University, China
